# The North-West London Hospital

**Published:** 1908-05-02

**Authors:** Alfred Craske

**Affiliations:** Secretary. North-West London Hospital, Kentish Town Road


### Editor's Letter-Box.

### THE NORTH-WEST LONDON HOSPITAL.

(*To the Editor of* The Hospital.)

Sir,—The amalgamation of the Hampstead General Hospital with the North-West London Hospital is now an accomplished fact. There is, however, a great deal of misunderstanding as to what will be the effect of this scheme on the future of the North-West London Hospital, which, for the past thirty years, has done a splendid work in a densely populated district. It is commonly reported that this hospital is now practically wiped out.

To correct this erroneous idea I crave your indulgence to explain that far from such being the case, this hospital will be greatly benefited and strengthened by the amalgamation. The work here has been carried on under serious disadvantages. For instance, some of the wards, consisting of small rooms in private houses, were, to say the least, ill-adapted for the purpose, and the King's Fund made it unmistakably clear that their generous support could not be continued under such conditions. To build a new hospital would have cost at least £80,000—a financial undertaking which my Committee were not prepared to face.

Owing to the good offices of the King's Fund, coupled with the promise of munificent help, the amalgamation scheme has now been effected, whereby we at the North-West London Hospital get all the benefit of a splendidly equipped modern hospital without the outlay of a penny in bricks and mortar. Among other advantages are these. Instead of fifty beds as hitherto, there will now be ninety beds available for our patients, while the out-patients' work will be continued in the Kentish Town Road under greatly improved conditions, where there is a resident staff of medical officers and nurses, and where cases of accident and emergency will be received. My Committee confidently hope that the governors and subscribers of the North-West London Hospital and all its old friends will generously support a scheme which under such improved conditions will continue this valuable work. Subscriptions and donations will be thankfully received and acknowledged by

Your faithful servant,

Alfred Craske, Secretary.

North-West London Hospital,
Kentish Town Road, April 22, 1908.

